# Neurophenotypes of COVID-19: risk factors and recovery trajectories

**DOI:** 10.21203/rs.3.rs-2363210/v1

**Published:** 2022-12-21

**Authors:** Divya Prabhakaran, Gregory Day, Bala Munipalli, Beth Rush, Lauren Pudalov, Shehzad Niazi, Emily Brennan, Harry Powers, Ravi Durvasula, Arjun Athreya, Karen Blackmon

**Affiliations:** Mayo Clinic

## Abstract

Coronavirus disease 2019 (COVID-19) infection is associated with risk of persistent neurocognitive and neuropsychiatric complications, termed “long COVID”. It is unclear whether the neuropsychological manifestations of COVID-19 present as a uniform syndrome or as distinct neurophenotypes with differing risk factors and recovery trajectories. We examined post-acute outcomes following SARS-CoV-2 infection in 205 patients recruited from inpatient and outpatient populations, using an unsupervised machine learning cluster analysis, with objective and subjective neuropsychological measures as input features. This resulted in three distinct post-COVID clusters. In the largest cluster (69%), cognitive functions were within normal limits (“normal cognition” neurophenotype), although mild subjective attention and memory complaints were reported. Cognitive impairment was present in the remaining 31% of the sample but clustered into two differentially impaired groups. In 16% of participants, memory deficits, slowed processed speed, and fatigue were predominant. Risk factors for membership in the “memory-speed impaired” neurophenotype included anosmia and more severe COVID-19 infection. In the remaining 15% of participants, executive dysfunction was predominant. Risk factors for membership in this milder “dysexecutive” neurophenotype included disease-nonspecific factors such as neighborhood deprivation and obesity. Recovery trajectories at 6-month follow-up differed across neurophenotypes, with the normal cognition group showing stability, the dysexecutive group showing improvement, and the memory-speed impaired group showing persistent processing speed deficits and fatigue, as well as worse functional outcomes. These results indicate that there are multiple post-acute neurophenotypes of long COVID, with different etiological pathways and recovery trajectories. This information may inform phenotype-specific approaches to treatment.

## Introduction

Cognitive and psychiatric symptoms are among the most common, persistent, and disabling consequences of COVID-19 [1-3]. Post-COVID cognitive impairment is reported in 20 to 25% of patients during the post-acute recovery phase [4] and in 15 to 35% of patients during the chronic “long COVID” phase [5]. Self-reported cognitive complaints include problems with concentration, memory, and slowed thinking [3-4], commonly referred to as “brain fog”. Objective neuropsychological assessment shows predominant impairment in attention, executive functioning, and memory, with relative preservation of language and visuospatial functions [6]. The most common post-COVID neuropsychiatric manifestations (new onset and chronic) include fatigue, anxiety, depression, insomnia, and posttraumatic stress disorder (PTSD) [7]. At this point, it remains unclear whether these heterogenous cognitive and psychiatric sequelae are uniformly elevated or clustered into distinct post-COVID neurophenotypes. This is important to determine, as different post-acute neurophenotypic presentations could point backwards to different etiological factors and forwards to different recovery trajectories.

Several different mechanisms may contribute to persistent cognitive and psychiatric sequalae following COVID-19 infection. These include neuroinvasion of SARS-CoV-2 into brain or neuroepithelial tissue and indirect damage from respiratory failure (hypoxic-ischemic effects), stroke, multi-organ system dysfunction, inflammasome activation, and complex pandemic-related psychosocial factors, such as social isolation, fear, and altered sleep, diet, exercise, and other health behaviors [8-11]. Risk factors for long COVID are heterogenous. Higher COVID-19 disease severity is associated with greater risk of long COVID [12] but even mild to moderate infections can increase risk of adverse neuropsychiatric outcomes [1, 13]. Cognitive profiles vary across studies, and it remains unclear whether disease-specific features (i.e., acute COVID-19 severity, presence of anosmia, encephalopathy, stroke, etc.) and disease-nonspecific features (sociodemographics, medical comorbidities, etc.) differentially contribute to distinct post-acute neuropsychological profiles. Given the variety of explanatory factors and ways in which they may interact, it is likely that the neuropsychological manifestations of long COVID present as a multiform rather than a uniform cognitive syndrome.

Recognizing this, we examined objective and subjective neuropsychological outcomes of SARS-CoV-2 infection in ambulatory and hospitalized patients using standardized assessment procedures across successive COVID-19 variant waves. We sought to characterize multivariate neuropsychological outcome clusters (i.e., “neurophenotypes”), identify their associative features, and examine recovery trajectories. Participants were assessed following their acute recovery and 6-months later with symptom inventories and a computerized cognitive testing platform. We hypothesized that neurocognitive and neuropsychiatric sequelae of COVID-19 would cluster into distinct neurophenotypes, each related to unique disease-specific and non-specific factors, and each differing in their longitudinal recovery trajectories.

## Materials And Methods

### Participants

The Mayo Clinic Institutional Review Board approved this prospective longitudinal cohort study. Informed consent was obtained electronically from all participants. Participants were recruited between July 2020 and February 2022 from a hospital-wide registry of Mayo Clinic Florida patients who tested positive for SARS-CoV-2 infection. All participants were ≥ 18 years of age and had no history of major neurocognitive disorders. Initial outcome assessment occurred within 12 weeks of PCR-confirmed infection (post-acute recovery stage). Follow-up assessments occurred 6 months later (chronic recovery stage). Participation required access to a computer for consent, test, and survey completion. All participants were emailed a link to complete assessments and respond to questionnaires.

Participant demographics and medical history were abstracted from the electronic health record (EHR) at time of initial post-acute outcome assessment. Medical comorbidities were summarized with the Elixhauser Van-Walraven Index (EVCI). The EVCI includes 31 common chronic medical conditions and was calculated from EHR data up to 1 year prior to PCR-positive test date [14]. Higher scores indicate a greater number of medical comorbidities. Vascular risk factors (history of smoking, diabetes, hypertension, and obesity) were identified by ICD-10 diagnostic codes in the patient’s EHR [15] and separately summarized due to the specialized role that vascular risk factors play in adverse COVID-19 outcomes [16]. Sociodemographic disadvantage was summarized with the 2019 Area Deprivation Index (ADI). ADI scores for each participant were retrieved from the University of Wisconsin-Madison’s Neighborhood Atlas [17], which derives national percentile rankings of socioeconomic disadvantage at the (US) Census Block Group neighborhood level from 1 (least disadvantaged) to 100 (most disadvantaged) based on unemployment rates, poverty, education, and housing [18].

COVID-19 disease severity was determined by an infectious disease specialist (HRP), using the National Institute of Allergy and Infection Disease Ordinal Scale (NIAID-OS) [19], with lower scores indicating higher illness severity. The following COVID-19 disease-specific factors were assessed: hospitalization status (ambulatory versus hospitalized), symptom status (symptomatic versus asymptomatic), and presence of anosmia (yes/no). Vaccination status was coded at 3 levels: vaccine not available (prior to FDA approval); unvaccinated (vaccine FDA approved but participant remained unvaccinated); and vaccinated. Finally, COVID-19 variant type was estimated from peak variant prevalence data at covariants.org [20] by test region (Florida) and time period (binned in 2-week intervals).

### Neurocognitive Assessment

We assessed objective cognitive performance with the CNS-Vital Signs (CNSVS) computerized neurocognitive assessment during the post-acute and chronic stages of recovery. The CNSVS includes the neurocognitive domains of verbal memory (immediate and delayed word recognition), visual memory (immediate and delayed design recognition), psychomotor speed (finger tapping and symbol digit coding tests), reaction time (averaged across Stroop congruent and incongruent trials), complex attention (sum of errors from continuous performance, shifting attention, and Stroop tests), and cognitive flexibility (correct responses on the shifting attention test minus the number of errors on the shifting attention test and Stroop test), as previously described [21]. Domain scores were age-adjusted by comparison to a normative reference group (mean = 100, standard deviation = 15), which was collected by the test publisher prior to the COVID-pandemic [21]. Classification of impairment (< 9th percentile) was based on the American Academy of Clinical Neuropsychology consensus conference statement on uniform labeling of performance test scores [22]. CNSVS includes embedded validity indicators, which show overall high accuracy in identifying intentional attempts to underperform [23]. Scores that were flagged as invalid were removed prior to analyses.

### Neuropsychiatric Symptom Inventories

We assessed subjective neuropsychological symptoms with the Neuropsych Questionnaire-45 (NPQ-45) during the post-acute and chronic stage of recovery. The NPQ-45 is a self-report symptom inventory that probes 12 neuropsychiatric symptom domains [24]. Scores from the following domains were summed and scaled as minimal (0–74), mild (75–149), or moderate to severe (150–300): attention (e.g., concentration difficulties), memory (e.g., forgetfulness), anxiety (e.g., nervousness, restlessness), depression (e.g., feeling discouraged, lack of interest), fatigue (e.g., low energy, weakness), and pain (e.g., headaches, muscle pain). During the chronic recovery stage, participants also completed the Medical Outcomes Survey (MOS-SF 36) [25] and a posttraumatic stress disorder (PTSD) checklist (PCL-C 17) [26].

### Data Imputation

Multivariate Imputation via Chained Equations (MICE) with predictive mean matching for 5 imputations and 50 iterations [27] was used to complete missing data from the post-acute neurocognitive assessment and neuropsychiatric symptom inventories.

### Unsupervised Machine Learning: K-means Clustering

Unsupervised machine learning methods were used to perform cluster analyses, given that they allow for inference of subgroups (referred to as clusters) within a dataset. Algorithms in unsupervised learning strive to maximize inter-cluster separation and minimize separation among samples within a cluster. Objective and subjective neuropsychological measures collected during the post-acute illness stage were used as input features in a K-means clustering analysis. No domain bias was applied to inputs. The optimal number of k-means clusters was determined with the elbow method [28]. Model fitting was performed with NbClust [29], implemented in R studio build 492 [30], with R v4.1.1 [31]. IBM SPSS Statistics 27 was used to perform K-means clustering and remaining data analyses.

### Cross-Sectional and Longitudinal Cluster Features

Clinical and sociodemographic factors associated with cluster membership were identified through Pearson χ2 and analysis of variance (ANOVA) statistical tests for parametric data and Kruskal-Wallis tests for non-parametric data. Normality was determined by kurtosis, skewness, and Shapiro-Wilk tests. Within-subjects repeated-measures ANOVA was used to examine changes in neurocognitive performance across two timepoints (1 and 6 months) by cluster. Significance was set at p < 0.05. Post-hoc analysis was conducted using Bonferroni correction to counteract Type I errors. Significance was set at p < 0.05.

## Results

During the study period, 205 participants (171 ambulatory, 34 hospitalized) completed post-acute neuropsychological outcome assessments 5.7 (± 3.8 weeks) following positive laboratory confirmation of SARS-CoV-2 infection. Of these, 79 participants completed the 6-month outcome assessment (65 ambulatory, 14 hospitalized). Attrition analyses are described in Supplementary Materials and Methods.

### Post-Acute Neuropsychological Outcomes K-means Cluster Analysis

**Figure 1:**
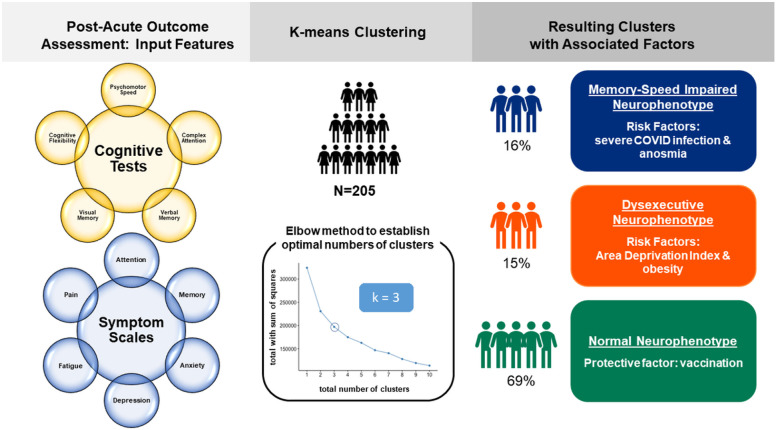
Unsupervised machine learning methods were used to perform cluster analyses on input features collected during the post-acute recovery stage in 205 patients with PCR-confirmed COVID-19 infections. Patients ranged in illness severity from asymptomatic to mild (ambulatory) to severe (hospitalized) cases. The optimal number of k-means clusters was determined with the elbow method. The resulting clusters were found to be associated with different disease-specific (anosmia, illness severity) and disease-nonspecific (obesity and area deprivation) risk factors.

All input features met a < 12% missingness threshold prior to MICE imputation. We determined with the elbow method that the optimal number of clusters was k = 3 (Fig. 1). Cluster 1 (N = 31) was characterized as “dysexecutive” due to impaired cluster centers for cognitive flexibility and complex attention (Supplementary Table 2). This dysexecutive cluster was also characterized by the presence of mild-to-moderate complaint severity for anxiety, attention, memory, fatigue, and pain. Cluster 2 (N = 32) was characterized as “memory-speed impaired” due to impaired cluster centers for verbal memory, psychomotor speed, and reaction time, as well as low average cluster centers for visual memory and cognitive flexibility. This memory-speed impaired cluster was also characterized by mild complaint severity for memory, attention, anxiety, depression, and pain, as well as moderate-severe complaint severity for fatigue. Cluster 3 was the largest cluster (N = 142) and was characterized as “normal cognition” due to cluster centers in the average/normal range for all cognitive domains. Notably, despite normal objective cognitive performance, participants in this cluster still reported mild complaint severity for anxiety, attention, memory, fatigue, and pain.

To compare percentages of cognitive impairment with other studies reported in the literature, we calculated the rates of subjective and objective cognitive impairment by cluster in the post-acute recovery stage (Supplementary Tables 3). Objective verbal memory impairment was present in 62.5% of the memory-speed impaired cluster, 6.5% of the dysexecutive cluster, and 9.4% of the normal cognition cluster. Subjective memory complaints (moderate-severe) were present in 56.3% of the memory-speed impaired cluster, 29% of the dysexecutive cluster, and 21.4% of the normal cognition cluster.

### Disease-specific Risk Factors for Cluster Membership

There were no significant associations between cluster membership and COVID-19 variant type (χ2 = 5.56, p = 0.24) or symptom status (χ2 = 0.42, p = 0.81). There was a marginal relationship between cluster membership and hospitalization status (χ2 = 5.9, p = 0.05), with the highest hospitalization rates in the memory-speed impaired cluster (31%). Cluster membership was associated with vaccination status (χ2 =11.64, p = 0.02); the normal cognition cluster had the highest vaccination rate (51%), while the memory-speed impaired cluster had the lowest vaccination rate (22%). Lack of vaccine availability at time of infection (i.e., infection prior to 12/17/20) was the most common reason why patients were unvaccinated across all 3 clusters. There was a strong association between cluster membership and anosmia (χ2 =12.02, p = 0.002), as well as NIAID scores (H (2) = 10.20, p = 0.006), with the memory-speed impaired cluster showing the highest rate of anosmia (70%) and lowest median NIAID scores (higher disease severity). Full results are presented in Table 1.

### Disease-nonspecific Risk factors for Cluster Membership

There were no differences in sex distribution (χ2 = 2.77, p = 0.25) or years of education (F = 0.78, p = 0.46) between clusters. Participant age differed across clusters (F = 8.25, p < 0.001), with the memory-speed impaired cluster showing the youngest mean age. Regarding medical risk factors, there were no associations between cluster membership and history of smoking (χ2 = 0.15, p = 0.93), hypertension (χ2 = 2.55, p = 0.28), diabetes (χ2 = 0.68, p = 0.71), or total EVCI score (H (2) = 0.68, p = 0.71). There was an association between cluster membership and obesity (χ2 = 8.82, p = 0.01) and ADI scores (H (2) = 6.49, p = 0.04). The dysexecutive cluster had the highest obesity rate (29%) while the normal cognition cluster had the lowest obesity rate (10%). ADI scores were highest in the dysexecutive cluster, indicating greater socioeconomic disadvantage. Full results are presented in Table 1.

### Recovery Trajectories

**Figure 2:**
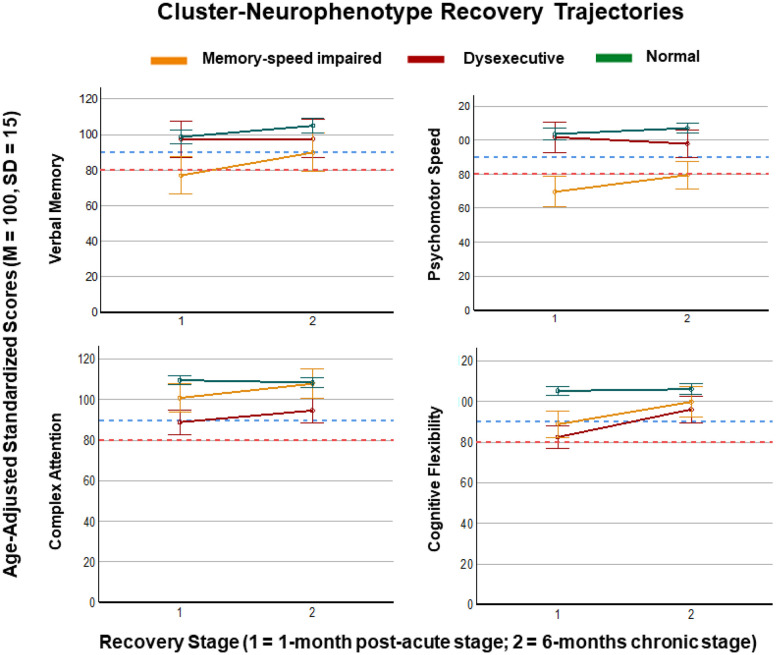
Estimated marginal means of neurocognitive recovery trajectories with 95% confidence intervals by cluster-neurophenotype (yellow=memory-speed impaired; red=dysexecutive; green=normal). Blue-dashed line indicates threshold for average performance, below red-dashed line indicates impairment. Between 80 and 90 (inclusive) is categorized as low average performance.

Seventy-nine of the original 205 participants (39%) completed outcome assessment in the 6-month chronic recovery stage (dysexecutive cluster 1: N = 9; memory-speed impaired cluster 2: N = 9; normal cognition cluster 3: N = 61). Results from repeated-measures ANOVA with disease stage (post-acute versus chronic) as a within-subjects factor and cluster membership (3 clusters) as a between-subjects factor showed a main effect of disease stage, with all groups showing improvement over time in verbal memory (F = 5.55; p = 0.021), complex attention (F = 6.60; p = 0.013), and cognitive flexibility (F = 31.90; p < 0.001). There was also a main effect of cluster membership, with the dysexecutive cluster showing lower cognitive flexibility (F = 19.45, p < 0.001) and complex attention (F = 18.32, p < 0.001) than the memory-speed impaired cluster and the normal cognition cluster, independent of time-point. The memory-speed impaired cluster showed lower verbal memory (F = 7.25, p < 0.001), psychomotor speed (F = 26.60, p < 0.001), and reaction time (F = 10.73, p < 0.001) than the dysexecutive and normal cognition clusters, independent of disease stage. Finally, there were several interactions between cluster membership and disease stage. Within the dysexecutive cluster, complex attention (F = 4.80, p = 0.01) and cognitive Flexibility (F = 11.69, p < 0.001) had steeper recovery trajectories in comparison to the memory-speed impaired and normal cognition clusters. The memory-speed impaired cluster showed a steeper recovery trajectory in psychomotor speed compared to the dysexecutive group (F = 4.06, p < 0.02) but their performance remained below both other clusters at the 6-month chronic disease stage (Fig. 2).

Analysis of subjective and objective impairment rates in each cluster at the post-acute and chronic timepoints showed that fatigue improved in the normal cognition cluster (χ2 = 6.61, p = 0.04), while the memory-speed impaired cluster continued to report moderate to severe fatigue (χ2 = 1.07, p = 0.59) in the chronic recovery stage. Approximately half of the memory-speed impaired cluster continued to report (66.7%) and show (44.4%) memory impairment in the chronic recovery stage, compared to smaller rates of persistent subjective and objective impairment in the dysexecutive and normal cognition clusters (Supplementary Table 4).

### Long-term Functional Outcomes

Comparison of functional outcomes between clusters at the 6-month chronic recovery stage revealed that the memory-speed impaired cluster had higher PTSD checklist (PCL-C 17) scores (H(2) = 7.39, p = 0.03) and worse functional outcomes (MOS-36 scores) compared to the other two clusters (Table 2), with particularly strong effects for physical functioning, general health, and health change (i.e., decline in health relative to one year ago).

## Discussion

In the current study, we extracted three distinct neurophenotypes from multivariate neuropsychological data collected in adults recovering from SARS-CoV-2 infection. Risk factors and recovery trajectories were distinct across neurophenotypes, validating the clinical utility of this approach. There were several important findings that can be used to guide evaluations of post-COVID patients and clinical trials of therapeutics designed to target the cognitive sequelae of long COVID.

First, most participants (69%) performed within normal limits on objective cognitive measures during the post-acute recovery stage. These participants were classified in the “normal cognition” cluster, although they did report mild severity inattention, fatigue, memory, and pain complaints. Such complaints are often sufficient to prompt evaluation in post-COVID care clinics [32], particularly if there is subjective experience of health change/decline. On average, this neurophenotype showed improvement in memory and psychomotor speed over time, although this may have been at least partially due to practice effects. Importantly, membership in this group predicted normal functional outcomes 6 months after SARS-CoV-2 infection, which is a point that can be used to counsel patients with mild post-COVID neuropsychiatric complaints who perform normally on objective cognitive testing.

Second, we found a rate of cognitive impairment (31%) among our participants that was consistent with that reported in the literature [4, 5]. Among the 31% of participants who showed cognitive impairment, there were two distinct clusters: a memory-speed impaired cluster and a dysexecutive cluster. This is consistent with the types of deficits that have been reported [6] but suggests two distinct patterns of impairment with different clinical implications.

Of these, the memory-speed impaired cluster can be considered the most severe neurophenotype. In addition to impaired performance on measures of verbal memory, psychomotor speed, and reaction time, there was also subtly reduced performance on measures of visual memory and cognitive flexibility. Individuals in this group reported the highest rates of subjective inattention, poor memory, and fatigue. Although there was encouraging evidence of multi-domain improvement over time, they exhibited persistent moderate-to-severe fatigue, memory and processing speed impairment, elevated PTSD symptom severity, and functional disability at 6-months follow-up. They reported the highest rates of health change (decline) over the past year. Although medical comorbidities can increase the risk of more severe COVID-19 infection and contribute to overall health decline, membership in this cluster was not associated with medical comorbidity status in the 1-year leading up to infection. Rather, risk factors included higher COVID-19 symptom severity, lower vaccination rate (largely due to the lack of vaccine availability at time of infection), and the presence of anosmia during acute infection, which are all disease-specific factors.

This raises the possibility that cognitive impairment in the memory-speed impaired neurophenotype may be due to pathologic mechanisms directly related to SARS-CoV-2 infection. Higher disease severity in COVID-19 reflects an increased need for respiratory support, which suggests that hypoxic-ischemic damage is an important etiological factor to consider [33, 34], especially as this is an established risk factor for memory impairment following critical illness in general [35] and COVID-19 specifically [34]. Direct and indirect neuroinvasion must also be considered. Post-mortem investigations of SARS-CoV-2 infected patients have shown neural invasion and cell death through infected astrocytes [8] in regions that are part of the suspected neural–mucosal CNS entry route [37] and are proximal to regions implicated by neuroimaging of living patients, such as the piriform cortex, parahippocampal gyrus, and orbitofrontal cortex [8, 38], all of which are known to support memory and neuropsychiatric functions. An increasing number of studies also establish the inflammatory consequences of COVID-19 within the central nervous system [39]. Biofluid biomarkers of astroglial activation (YKL-40) and pro-inflammatory cytokines (e.g., IL-1β, IL-6, IL-8, and TNF-α) distinguish cases from healthy uninfected controls [40], while markers of neuroaxonal loss (e.g., neurofilament light, total-tau) rise in proportion with disease severity, with higher levels identifying patients with worse outcomes at hospital discharge [41]. Collectively, these findings suggest that post-COVID cognitive sequelae in the memory-speed impaired cluster may arise from the combined direct and indirect effects of COVID-19 infection on the brain.

Surprisingly, younger individuals had a higher risk of membership in the memory-speed impairment cluster. This has two important implications. One is that the memory impairment in this group is unlikely to reflect unmasking of an incipient age-related neurodegenerative disease [42]. The second is that these are individuals who would be otherwise working, raising families; thus, persistent cognitive impairment in this cohort is likely to result in greater functional impairment, raising per capita and indirect costs of disability, similar to what has been documented in conventional brain injury groups [43]. For these young patients, early and intensive cognitive rehabilitation efforts are essential, not just for recovery and community integration, but for minimizing the financial impact of COVID-19 infection.

The dysexecutive neurophenotype was characterized by impairment in complex attention and cognitive flexibility. This was a milder neurophenotype that showed a steeper recovery trajectory. Subjective cognitive complaints were only slightly higher than what was reported by the normal cognition cluster. On average, domain scores for complex attention and cognitive flexibility improved to the normal range by 6 months. The base rates for impairment in complex attention dropped from 36–0% and for cognitive flexibility from 52–11%. However, attrition may have inflated improvements, as those who completed 6-month follow-up had higher baseline cognitive flexibility than those who were lost to follow-up. Risk factors for cluster membership included COVID-nonspecific factors such as neighborhood deprivation and obesity. Participants from communities with higher ADI scores are more likely to experience systemic disadvantage, potentially manifesting as reduced access to physical and mental healthcare, food insecurity, reduced exercise opportunities, more air pollution and unsafe housing, social discrimination, and increased worry about pandemic-related factors [44-46]. They are more likely to be concerned about the varied economic effects of the pandemic, school closures and coordination of work and childcare responsibilities, occupational exposure to the virus, access to and cost of healthcare, ability to socially distance, and concern for older family members potentially living in the same household, all stressors that could impact cognitive performance [47, 48]. Obesity is more common in areas of lower socioeconomic status [49], which suggests that these may not be independent risk factors.

### Treatment Considerations

Our findings emphasize differences and similarities across patients with long COVID symptoms. Post-acute neuropsychological profiles clustered into three distinct neurophenotypes, each of which was associated with distinct risk factors and recovery trajectories. These findings can inform phenotype-specific approaches to treatment, highlighting the need for different treatment approaches rather than a “one size fits all” response to post-COVID symptoms. This is important not only for prudent programmatic resource allocation and financial effect modelling within medical provider teams but also for minimizing out of pocket expenses incurred by patients. Importantly, we found that more than two-thirds of patients ascertained from a hospital registry do not have objective cognitive impairment. For many, inefficiencies in attention, memory, and speeded resolved within 6 months of infection. For the normal and dysexecutive neurophenotypes, reassurance and lifestyle counseling will likely be important to improve long-term wellness, along with public and private health initiatives to improve pandemic childcare policies, employee sick time policies, and healthcare access. Cognitive Behavioral Therapy (CBT) is also likely to provide benefit for those reporting persistent anxiety, depression, insomnia, and fatigue [50].

For the memory-speed neurophenotype, a comprehensive interdisciplinary rehabilitation approach that incorporates physical therapy, occupational therapy, nursing, and psychology may be particularly important, as has been demonstrated in comprehensive pain clinics [51, 52]. Realistic goal-setting, activity pacing, and empowered self-management of symptoms are essential components of therapy [53 Skilbeck 2022]. Targeted cognitive rehabilitation in long COVID patients has been shown to be effective for remediation of memory impairment [54]. Rehabilitative therapies can focus on recovery strategies but also on compensatory memory strategies to attenuate frustration and facilitate adjustment to life with memory dysfunction. Individualized recommendations from cognitive rehabilitation specialists can inform accommodations to support successful return to work, school, or community reintegration.

### Limitations

An important study limitation was high participant attrition rates. Although there were no significant differences in follow-up rates by cluster, the mild and severe neurophenotypes had smaller sample sizes compared to the normal cluster. Disproportionate cluster size was not predicted in advance due to the unknown nature of the disease. Results provide valuable information for prospective study planning. Larger cohorts will be necessary to obtain sufficient sample size for the dysexecutive and memory-speed impaired neurophenotypes in future longitudinal outcome investigations. An additional limitation is that we did not evaluate whether participants received formal interventions or therapeutics in the time between post-acute and chronic assessments; therefore, we cannot attribute recovery to the “natural course” of the disease.

## Conclusion

The neurologic manifestations of long COVID present as distinct neurophenotypes with different risk factors and recovery trajectories. Future efforts should seek to replicate these neurophenotypes and their associated features in independent samples. It will be important to directly test whether the efficacy of various post-COVID therapeutics differs across neurophenotypes, given the high likelihood that different etiological factors contribute to post-COVID cognitive sequelae and influence recovery.

## Figures and Tables

**Table 1:** Neurophenotypes: associated features and risk factors.

Feature	Cluster 1 Dysexecutive (N = 31)	Cluster 2 Memory-Speed Impaired (N = 32)	Cluster 3 – Normal Cognition (N = 142)	*p-* *value*
**Age**, M (SD), y	50.06_a_ (13.12)	47.22_a_ (14.49)	56.81_b_ (13.50)	< 0.001*
**Sex**, n (%)				0.25
Female	22 (71.0%)_a_	21 (65.6%) _a_	80 (56.3%) _a_	
Male	9 (29.0%) _a_	11 (34.4%) _a_	62 (43.7%) _a_	
**Covid Variant Type**, n (%)				0.24
Initial/Alpha	14 (45.2%) _a_	21 (65.6%) _a_	63 (44.4%) _a_	
Delta	9 (29.0%) _a_	5 (15.6%) _a_	47 (33.1%) _a_	
Omicron	8 (25.8%) _a_	6 (18.8%) _a_	32 (22.5%) _a_	
**Hospitalization Status**, n (%)				0.05
Ambulatory	27 (87.1%)_a_	22 (68.8%)_a_	122 (85.9%)_a_	
Hospitalized	4 (12.9%)_a_	10 (31.3%)_a_	20 (14.1%)_a_	
**NIAID Score**, median (range)	8 (3, 8)_a_	7 (3, 8)_b_	8 (3, 8)_a_	0.006*
**Anosmia**, n (%)				0.002*
No Anosmia	19 (63.3%)_a_	9 (30.0%)_b_	87 (64.0%)_a_	
Anosmia	11 (36.7%)_a_	21 (70.0%)_b_	49 (36.0%)_a_	
**Vaccination Status**, n (%)				0.02*
Vaccine Unavailable	14 (45.2%)_a,b_	21 (65.6%)_a_	59 (42.8%)_b_	
Unvaccinated	5 (16.1%)_a,b_	4 (12.5%)_a_	8 (5.8%)_b_	
Vaccinated	12 (38.7%)_a,b_	7 (21.9%)_a_	71 (51.4%)_b_	
**Area Deprivation Index (ADI)**, median (range)	37 (4, 80)_a_	31 (8, 94)_a,b_	30 (2, 94)_b_	0.03*
**Elixhauser van-Walraven Index (EVCI)**, median (range)	0 (−4, 21)_a_	0 (−4, 31)_a_	0 (−4, 27)_a_	0.86
**History of Smoking**, n (%)				0.93
Yes	7 (25.0%) _a_	6 (20.7%) _a_	30 (22.6%) _a_	
No	21 (75.0%) _a_	23 (79.3%) _a_	103 (77.4%)_a_	
**Diabetes**, n (%)				0.71
Yes	2 (6.5%) _a_	4 (12.5%) _a_	13 (9.3%) _a_	
No	29 (93.5%) _a_	28 (87.5%) _a_	127 (90.7%) _a_	
**Hypertension**, n (%)				0.28
Yes	8 (25.8%) _a_	14 (43.8%) _a_	44 (31.4%) _a_	
No	23 (74.2%) _a_	18 (56.3%) _a_	96 (68.6%) _a_	
**Obesity**, n (%)				0.01*
Yes	9 (29.0%)_a_	7 (21.9%)_a,b_	14 (10.0%)_b_	
No	22 (71.0%)_a_	25 (78.1%)_a,b_	126 (90.0%)_b_	

National Institute of Allergy and Infectious Diseases (NIAID) severity scale: 8) Death; 7) Hospitalized, on invasive mechanical ventilation or extracorporeal membrane oxygenation (ECMO); 6) Hospitalized, on non-invasive ventilation or high flow oxygen devices; 5) Hospitalized, requiring supplemental oxygen; 4) Hospitalized, not requiring supplemental oxygen - requiring ongoing medical care (COVID-19 related or otherwise); 3) Hospitalized, not requiring supplemental oxygen - no longer requires ongoing medical care; 2) Not hospitalized, limitation on activities and/or requiring home oxygen; 1) Not hospitalized, no limitations on activities.

Area Deprivation Index (ADI): Socioeconomic disadvantage at the (US) Census Block Group neighborhood level ranging from 1 (least disadvantaged) to 100 (most disadvantaged) based on unemployment rates, poverty, education, and housing.

Elixhauser van-Walraven Index (EVCI): Summary index of 31 common chronic medical conditions abstracted from the electronic health record up to 1 year prior to PCR-positive test date.

Cluster columns not sharing subscripts indicate mean or median differs significantly at p < 0.05 as indicated by Bonferroni correction.

**Table 2:** Neurophenotypes: 6-month functional outcomes

	Cluster 1 - Dysexecutive Function (N = 9)	Cluster 2 - Memory- Speed Impaired (N = 9)	Cluster 3 – Normal Cognition (N = 61)	*p-* *value*
**Medical Outcomes Survey (MOS SF-36)**, M (SD)				
**Physical functioning**	109.33 (9.42)_a_	88.67 (11.46)_b_	105.78 (11.14)_a_	0.002*
**Role functioning/physical**	109.00 (12.28)_ab_	90.00 (15.59)_a_	107.24 (13.71)_b_	0.01*
**Role functioning/emotional**	104.56 (12.42)_a_	99.22 (17.92)_a_	105.20 (13.59)_a_	0.47
**Energy/fatigue**	102.67 (11.57)_ab_	86.22 (19.65)_b_	102.17 (16.38)_a_	0.03*
**Emotional well-being**	103.22 (8.26)_a_	94.67 (16.47)_a_	104.76 (11.84)_a_	0.07
**Social functioning**	105.56 (11.78)_a_	89.00 (17.56)_b_	103.42 (12.31)_a_	0.02*
**Pain**	104.44 (12.33)_a_	87.22 (18.97)_a_	102.32 (12.40)_a_	< 0.05*
**General health**	105.67 (12.64)_a_	80.78 (18.71)_b_	106.36 (17.13)_a_	< 0.001*
**Health change**	101.22 (14.33)_a_	79.78 (14.85)_b_	95.15 (14.05)_a_	0.005*
**PTSD PCL-17**, median (range)	20 (17–37)_ab_	27 (19–65)_b_	20 (17–59)_a_	0.03*

MOS SF-36 scores are standardized to mean of 100 (SD = 15) based on comparison to a U.S. normative reference group that ranges in age from 18–94 years [25]. Lower scores indicate greater functional disability.

The PTSD PCL-17 contains 17 items that are summed as a severity score (1 = not at all, 5 = extremely) from a range of 17–85, with higher scores indicated greater PTSD symptom severity. Scores greater than 29 indicate moderate to severe PTSD and cut-off scores ranging from 30–50 have been used to define PTSD in prior research studies [55].

Cluster columns not sharing subscripts indicate mean or median differs significantly at p < 0.05 as indicated by Bonferroni correction.
